# Supplementary figures and images for: A lightweight intrusion detection method for IoT based on deep learning and dynamic quantization
Source: PeerJ Comput Sci. 2023 Sep 22;9:e1569. doi: 10.7717/peerj-cs.1569 (PMC10557502; doi:10.7717/peerj-cs.1569)

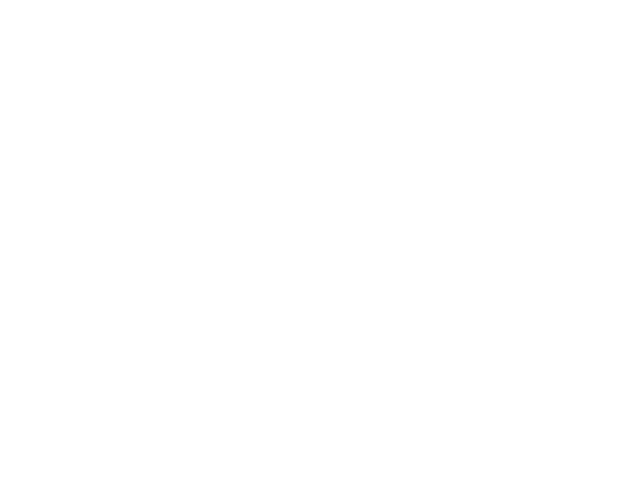

Supplement: Supplemental Information 1 [file peerj-cs-09-1569-s001.zip › source code/CIC IDS2017 experiment/contrast experiment/demo.jpg]
